# Implementation research to develop an optimized delivery model for effective implementation of evidence-based interventions to reduce stillbirth in India: A study protocol

**DOI:** 10.1371/journal.pone.0316027

**Published:** 2025-02-10

**Authors:** Gadapani Barsha Pathak, Reema Mukherjee, Vani Kandpal, Abhishek Agarwal, Prem Mony, Maryann Washington, Sarmila Mazumder, N. K. Arora

**Affiliations:** 1 Society for Applied Studies, New Delhi, India; 2 Division of Reproductive, Child Health and Nutrition, Indian Council of Medical Research, New Delhi, India; 3 Department of Research, The INCLEN Trust, New Delhi, India; 4 Division of Epidemiology, Biostatistics and Population Health, St. John’s Research Institute, Bangalore, Karnataka, India; Public Library of Science, UNITED KINGDOM OF GREAT BRITAIN AND NORTHERN IRELAND

## Abstract

**Background:**

Stillbirth remains a public health concern in India, despite a decrease in reported rates. Inconsistent data collection hampers clear understanding the burden of stillbirth, and interventions for its reduction are scattered across various national programs. This research aims to explore effective strategies to improve the delivery and uptake of high-quality antenatal and intrapartum care services which have the potential to reduce stillbirth rates in various states of India.

**Methods:**

This mixed-method, multi-site study in India will be conducted in three phases: Phase 1: Formative Phase; Phase 2: Development of a Comprehensive Package for Stillbirth Reduction and Optimization of the implementation model and Phase 3: Scale-up of comprehensive package and monitoring of optimized strategy/model. Participants will include pregnant women, women who have recently delivered, family members, respectable community members, healthcare workers and staff, state and district health authorities. The effectiveness of intervention package and optimized implementation model in reducing stillbirth will be evaluated using a pre-post quasi-experimental design. The burden of stillbirth will be estimated through community survey, recording pregnancy outcomes for women who have delivered within the past one year. Various methods including semi-structured questionnaires, verbal autopsies, and in-depth qualitative interview guides, review of clinical case sheets will be used to assess the causes of stillbirth. Additionally, government health facilities will be assessed and strengthened over study period. This study will utilize implementation science theories, models, and frameworks (TMF), including the Consolidated Framework for Implementation Research (CFIR) to identify barriers and facilitators, and the evaluative TMF of RE-AIM (Reach Effectiveness Adoption Implementation and maintenance) to monitor the optimized model. The primary outcome is the development of a scalable, sustainable model of intervention package and delivery strategies to reduce stillbirths. The secondary outcome includes a robust estimation of burden, timing, and risk factors of stillbirths across all study sites. Certain sites will conduct an economic evaluation to assess the incremental cost of implementing comprehensive packages using the optimized implementation model.

**Discussion:**

This innovative study addresses a critical public health gap in context of stillbirth reduction in India. Integrating proven interventions with real-world implementation challenges across diverse regions, this project aims to develop a comprehensive and replicable model. If successful, this model can significantly improve stillbirth prevention in low-resource settings.

**Trial registration:**

ClinicalTrials.gov CTRI-2024/07/069796.

## Introduction

Historically, the primary focus in India and other low- and middle-income countries (LMICs) has been on reducing under-five mortality. Substantial global progress have been made in reducing childhood mortality since 1990, with the total number of under-five deaths worldwide decreasing from 12.8 million in 1990 to 4.9 million in 2022 [1]. This achievement has been driven by efforts to tackle the predominant causes of child mortality, such as vaccine-preventable diseases, acute respiratory infections, diarrheal diseases, malaria, malnutrition, and tuberculosis. However, the reduction in neonatal mortality has not matched the pace of decline in post-neonatal under-five mortality. From 1990 to 2022, the decline in neonatal mortality has been slower [1], prompting a shift in focus to neonatal mortality reduction both globally and in India. Despite the increased focus on neonatal mortality, stillbirths remain a “neglected tragedy” in public health programs. They have been largely overlooked by governments, public health officials, and, until recently, international organizations, despite evidence showing that half of all stillbirths are preventable. The recent Every Newborn Action Plan (ENAP) has set a global target for countries to achieve a stillbirth rate of 10 or fewer per 1000 total births by 2035, aiming also to close equity gaps [2]. India’s Newborn Action Plan (INAP) echoes this global goal [3].

Stillbirth rate (SBR) varies considerably between different sources in India: the Sample Registration System (SRS) 2020 reports 3 per 1,000 births [4] and the Health Management Information System (HMIS) 2019-20 reports 12.4 per 1,000 births [5]. On the other hand, National Family Health Survey-5 indicates a stillbirth rate of 9.7 per 1,000 births [6], which is 3 times higher than the SRS rate. These differences point to issues with stillbirth definitions, gestation period documentation, and the classification of miscarriages and abortions, leading to underreporting in the SRS [7].Globally, evidence shows that stillbirths can be prevented through interventions focused on antenatal and intrapartum care, including nutrition, infection prevention, and management of medical conditions of pregnancy [8–10]. These interventions have been embedded into various national programs. In India, they are scattered across initiatives like the Newborn Action Plan, Janani Suraksha Yojana (JSY), Janani Shishu Suraksha Karyakram (JSSK), Pradhan Mantri Surakshit Matritva Abhiyan (PMSMA), and Labour Room Quality Improvement Initiative (LAQSHYA). However, a key challenge is expanding coverage, which has hindered the full potential of these programs in effectively reducing stillbirths.

Comprehensive research is needed to understand the medical, socio-economic, and environmental factors contributing to stillbirth. Additionally, it is important to investigate the reasons behind the low uptake of interventions in national programs aimed at promoting healthy pregnancy outcomes. Once these factors are understood, effective implementation strategies must be developed to increase coverage of these interventions, ensuring they are delivered in a coordinated and integrated manner within existing healthcare programs. This approach involves consolidating the currently fragmented interventions into a cohesive package, with strategies that address the identified determinants effectively. Along side delivering these interventions, establishing a robust evaluation framework is essential to monitor effectiveness and ensure desired outcomes. This evaluation will also incorporate key Implementation Research components, assessing contextual factors from the CFIR that influence implementation, as well as mediators and moderators affecting adoption. Additionally, it will identify organizational and contextual factors for long-term sustainability, ensuring the intervention package is impactful, scalable, and sustainable across diverse healthcare settings.

Hence this study will try to answer the question -What are the most effective strategies to improve the delivery and uptake of high-quality antenatal and intrapartum care services to reduce stillbirth rates?

### Aim

This implementation research aims to identify effective strategies to improve the delivery and uptake of evidence-based antenatal and intrapartum care, targeting stillbirth reduction. The study will develop, co-design, implement, and evaluate a feasible, scalable intervention model to reduce SBR across seven geographically and culturally diverse regions in India, including rural, peri-urban, and tribal areas.

### Objectives of the study

The primary objective is a) to develop, design and implement a comprehensive package of interventions and effective delivery strategies to improve coverage and process indicators which have the potential to reduce stillbirths and b) to estimate burden, timing and associated risk factors. (c)Monitoring the changes in the stillbirth rate during the study period will constitute the secondary outcome in certain sites.

## Methodology

This implementation research will employ a mixed-method approach to scale up an optimized intervention package for reducing stillbirths. Based on formative research and secondary data, a context-specific package will be developed and tested in seven geographically and culturally diverse sites. The implementation strategies will be optimized through iterative cycles involving concurrent implementation, evaluation of coverage and quality (primarily using quantitative methods), and refinement based on program learning derived from both quantitative and qualitative feedback. Periodic meetings, led by government authorities, will provide opportunities to reflect on the processes, share lessons learned, and continuously revise and improve the intervention package and implementation model.

### Study design, population, and setting

This study follows a multi-centric pre-post quasi-experimental design across seven sites. It utilizes both qualitative and quantitative data collection to assess contextual factors such as cultural and social perceptions and beliefs. Baseline data will be gathered to evaluate the performance and coverage of existing antepartum and intrapartum interventions within the healthcare system. Data will be collected through house-to-house cross-sectional surveys and direct observations of antenatal and intrapartum care practices in facilities, as well as socio-behavioral change communication (SBCC) activities in the community and healthcare facilities. Additionally, baseline assessments of healthcare facilities and the competency of healthcare providers will be conducted to identify gaps that need to be addressed for effective implementation.

Following the implementation of the comprehensive intervention package, an end-line cross-sectional survey will evaluate changes in key indicators. By comparing pre- and post-intervention data, the potential impact on stillbirth reduction will be assessed. Concurrent surveys during the optimization phase will gather additional data on variables that may contribute to this reduction.

Since the implementation strategies focus on multiple levels—community, facility, and health system—the study population will encompass participants from all these levels. This includes pregnant women, women who have recently delivered, their partners and family members, healthcare workers, as well as district and state health department authorities. Health facility assessments, including health and wellness centers, primary and community health centers, sub-district hospitals, district hospitals, and private centers (site-Karnataka), will also be conducted, with a focus on the knowledge and skills of healthcare providers. Detailed information about the study sites is provided in the Table 1.We have utilized the Standards for Reporting Implementation Studies (STARi) [11] checklist along with SPIRIT checklist as a reporting standard for this study (Fig 1).

**Table 1:** Details of the selected sites for the Stillbirth reduction implementation research.

Characteristics	Bareilly District (Uttar Pradesh)	East Khasi Hills District (Meghalaya)	Sangrur District (Punjab)	Pune District (Maharashtra)	Udaipur District (Rajasthan)	Haveri District (Karnataka)	Palwal District (Haryana)
**State SBR (HMIS 2021-22)**	9.2	27.7	12.16	8.1	19.9	9.7	12
**Demography**	Peri-Urban	Tribal	Rural	Rural & urban	Mainly Rural	77% Rural	Rural
**Terrain**	Plain	Hilly	Plain	Plain & hills	Plain & rocky mountains	Plain& hills	Plain
**Study blocks/Sub-district divisions**	4 Blocks	5 Blocks	4 Blocks	3 Blocks	3 Blocks	3 Taluka	5 Blocks

# SBR calculated based on HMIS (2021–22) (10).

**Fig 1:**
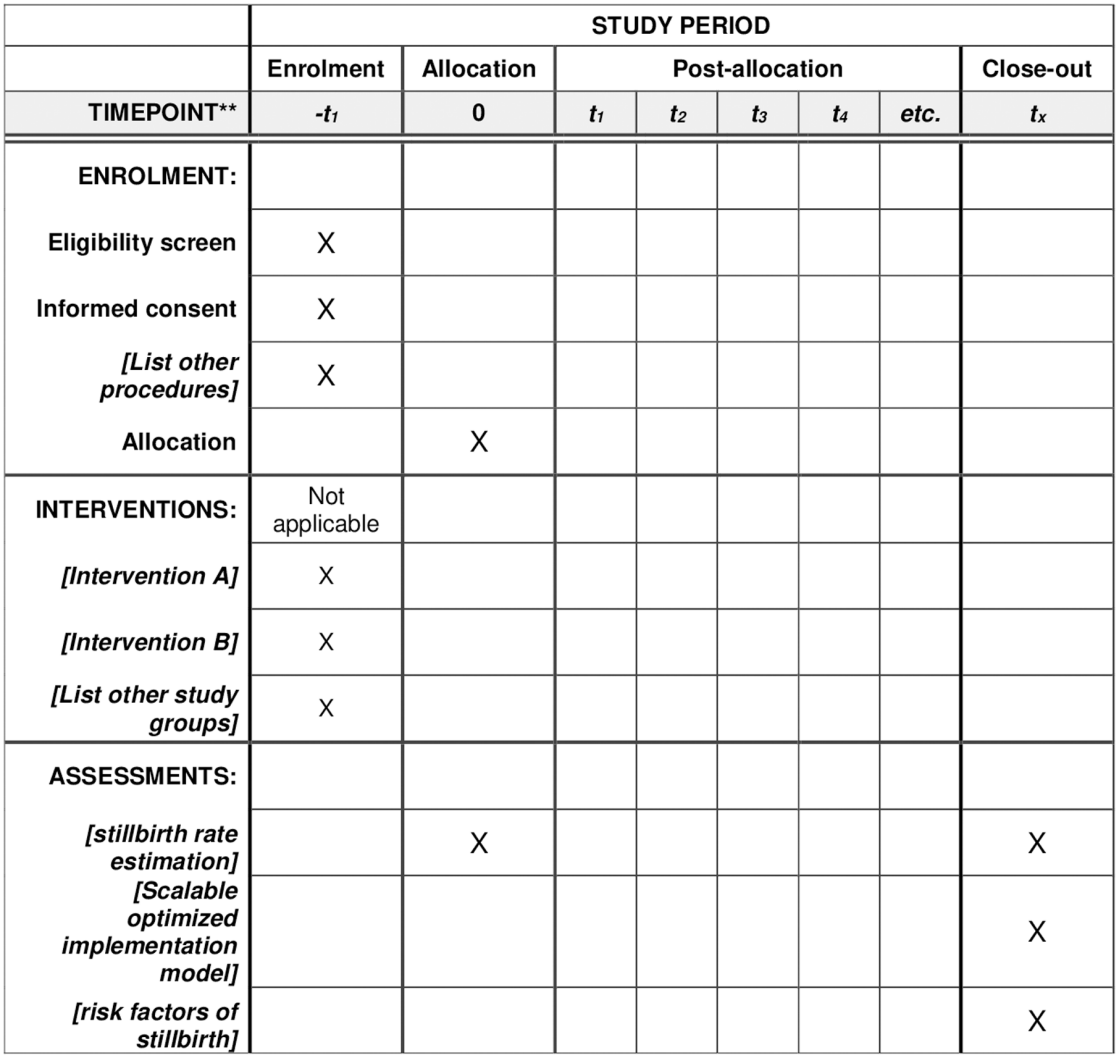
SPIRIT checklist for implementation research to develop an optimized model of interventions to reduce stillbirths.

### Sample size and sampling

The study will be conducted in seven districts, each with a population of approximately one million, across rural Haryana, Karnataka, Punjab, peri-urban Uttar Pradesh, and tribal regions of Meghalaya, Maharashtra, and Rajasthan. Sample size for each objective is different and describe below.

(a) **Stillbirth rate estimation:** To estimate the prevalence of stillbirth rate (SBR) at baseline and at the end of the study, sample sizes were computed separately for both time points, rather than for changes. Given that estimates of SBR in India tend to underestimate actual rates, there are no reliable data sources available for precise sample size estimation. To account for the stillbirth burden, sample sizes were calculated for sites with SBRs ranging from 10 to 25 per 1,000 births, using a relative precision of 30-35%, a design effect of 1.5, and a 95% confidence interval. A sample size of 3,000 was determined to be sufficient to estimate SBR accurately at baseline across all sites. The sample will be drawn from villages within selected blocks using a Population Proportionate to Size (PPS) strategy, recruiting women who delivered within the past year. For the endline, aiming for a single-digit SBR target of 9 per 1,000 births (the upper limit of single digits), a sample size of 4,000 per site was determined to be appropriate to accurately capture SBR.
(b) **Coverage of antenatal and intrapartum interventions targeting stillbirths:** To estimate the coverage of antenatal and intrapartum interventions aimed at reducing stillbirths, a sample size of 900 women has been calculated to target 80% coverage of a comprehensive package of quality services during both the antenatal and intrapartum periods. Women will be interviewed either before discharge from a healthcare facility after delivery or through a community survey of those who delivered within the last three months, at both baseline and endline. Additionally, clinical records, the Mother and Child Tracking Card, and ANC records will be reviewed during the interview to verify the care received. The sample size calculation assumes 80% coverage, with a 4% margin of error, a 95% confidence interval, and a design effect of 1.5. Eligible women must have lived in the study area for more than six months. A two-stage Population Proportion to Size (PPS) strategy will be used to recruit participants from villages within selected blocks. The coverage will be assessed at baseline and again at end line after implementing the optimized delivery model to evaluate changes in intervention coverage.
(c) **Direct observation:** Purposive sampling will be used to conduct direct observations of 100 antenatal care services and 100 deliveries for intrapartum care. In addition, 50 each of Village Health and Nutrition Days (VHNDs) and Pradhan Mantri Surakshit Mantritva Abhiyan (PMSMA) days which are held monthly for high-risk pregnancies, will be observed to assess the services and counseling provided to pregnant women. Facilities within the catchment area, including sub-centres, primary health centres, and all high-load secondary health centres, district hospitals, and sub-district hospitals will also be assessed, along with their staff. The study will also aim to include private facilities in the area, although their participation will depend on obtaining consent to share data and implement the intervention package.
(d) **Qualitative data** will be collected through In-Depth Interviews (IDI), Focused Group Discussions (FGDs), and observation with community health care workers, health professionals and state and district level administrators.While some of the sites will use saturation method regarding sample size, few other sites would cover certain number of participants. Verbal autopsy will also be conducted among mothers/families who had stillbirth using WHO 2016 to examine misclassification.
(e) In addition to this, a few critical coverage and process indicators will be collected on concurrent basis, once in every 3-4 months.

### Conceptual framework

The conceptual framework guiding this study draws upon the Consolidated Framework for Implementation Research (CFIR) that considers the multifaceted aspects of implementing complex interventions in real-world contexts by considering dimensions like intervention characteristics, outer and inner settings, individuals involved, and the process of implementation. Informed by the CFIR, this study investigates the intricacies of interventions directed to prevent and/or reduce stillbirths in India, with a specific focus on the antenatal and intrapartum periods. Additionally, the RE-AIM framework, which focuses on dimensions such as reach and effectiveness at the individual level, adoption and implementation at various levels (staff, setting, system, or policy), and maintenance at both individual and organizational levels [12] will be employed to monitor the optimized implementation model, assessing reach, adoption, implementation fidelity, and maintenance to evaluate the interventions and its delivery strategies’ sustainability and long-term effects.

### Comprehensive intervention package

While deciding on the intervention components, we looked at the evidence on the risk factors for stillbirths that are amenable to intervention. Maternal or pregnancy characteristics/ conditions strongly associated with stillbirth include maternal anemia, hypertensive disorders of pregnancy, gestational diabetes milletus and history of previous stillbirth or abortions. Increasing maternal age, specifically age above 35 years, increased gestational weight gain especially in the third trimester is also associated with stillbirths [13,14]. While fetal risk factor most strongly associated with still birth is small for gestational age and preterm births [13]. Maternal health behavior of reduced number of antenatal visits, tobacco smoking and sleep position is also associated with stillbirths [13,15,16]. At a more structural or health system level reduced quality of antenatal care and intrapartum quality of care are also associated with stillbirths [17].The national programmes already incorporate intervention or services that target these risk factors. However, a key consideration is that these interventions, while embedded within existing programs, are dispersed, potentially diverting implementors from focused delivery and diminishing compliance. This study takes a distinctive approach by consolidating these interventions into a comprehensive package, emphasizing their collective impact on reducing stillbirth rates. By amalgamating health-related and nutrition-focused, intrapartum care and health system strengthening strategies, the study aims to improve the coverage of these interventions (Table 2) [16–28].

**Table 2:** Comprehensive package for reduction of stillbirth in seven sites in India.

Stage	Category	Intervention components
**Preconception and Antepartum**	**Health related interventions**	1. Early registration and a minimum of 4 ANC visits [reduced ANC visits leading to increased perinatal mortality; RR 1.14, 95% CI 1.00 to 1.31] [16]. 2. Antenatal screening and management for maternal morbidities like [a] Gestational Diabetes Milletus [reduction in perinatal mortality; RR 0.51, 95% CI 0.14–1.88], [17] [b] Gestational Hypertension [18,19] [c] Other high risk case especially previous history of stillbirth/ miscarriage [18] 3. Screening and appropriate treatment for anemia [18,20] 4. Detection and appropriate management of SGA pregnancies [18]
	**Nutrition-related Interventions**	1. Preconception folic acid [evidence of benefit [RR 0.38, 95% CI 0.29–0.51] [21] 2. Provision of balanced energy protein supplementation [Stillbirth; RR = 0.60, 95% CI: 0.39-0.94] [22]. 3. Iron and folic acid supplementation [23]
	**Counselling and Behavioral change**	1. Sleep Position [24] 2. Tobacco use [20] 3. Birth preparedness, and promotion of institutional delivery [25]. 4. Nutrition counseling. [22]
**Intrapartum**	**Care during labour.**	1. Evidence based intrapartum care [26] 2. Identification and Induction of Mothers with ≥ 41 Weeks Gestation [Fewer perinatal deaths; RR = 0.31; 95% CI: 0.11-0.88] [27]
**Health System Strengthening**	**Capacity building**	Training healthcare workers in various guidelines & programs related to antenatal & intrapartum care. [Lower stillbirth rate; adjusted OR = 0.69, 95% CI 0.57 to 0.83] [28]

The health-related interventions include: [a] Early registration and a minimum of four “quality” ANC visits, and [b] Antenatal screening and management for maternal conditions such as GDM, gestational hypertension, and other high-risk cases [especially increased maternal age, gestational weight gain, previous history of stillbirth]. The quality of ANC care will be defined to ensure that these components target the modifiable risk factors for stillbirth. This will include tracking gestational weight gain, screening and managing anemia and hypertensive disorders, detecting and appropriately treating high-risk pregnancies, and providing fetal monitoring through ultrasonography and fetal Doppler where necessary. Additionally, components like nutrition counseling, tobacco cessation, and guidance on sleep position will further ensure that all major modifiable risk factors are effectively addressed. The nutritional interventions will include mobilization of women towards ‘Take Home Rations’, nutrition counseling and anemia management.

Adherence to evidence based intrapartum care and labour room guidelines and related protocols will constitute intrapartum interventions. Training of healthcare workers, perinatal death audit and integration of HMIS and reporting will constitute interventions regarding health system strengthening.

Furthermore, an umbrella review is being conducted to identify national and international best practices for reducing stillbirths which may enhance the comprehensive intervention package. Notably, certain study sites may also introduce additional interventions such as pre-conceptional care and the use of Minimally Invasive Tissue Sampling [MITS] for identifying the causes of stillbirth However, including these effective interventions in the comprehensive stillbirth reduction package and their implementation will depend on the consensus of the respective state governments.

However, due to the short duration of the study, it is possible that the effectiveness of the package may not be evaluated. In addition to this, incremental cost analysis of implementing the optimized model of interventions will be performed in selected sites..

### Study phases

Guided by inherent domains and constructs of CFIR, the research study has three phases. CFIR will support in identification of the drivers and challenges of implementation, further helping in model optimization. The study aims to understand the burden, timing, and factors linked to stillbirths, and to develop a scalable, sustainable intervention package with innovative delivery strategies. Using implementation research principles, it seeks to achieve high population-level coverage of evidence-based interventions within existing health programs aimed at reducing stillbirths. The optimized model will be evaluated for its effectiveness in reducing stillbirth across some of the study sites by using a quasi-experimental pre-post design.

#### Phase 1: Formative research.

This project will begin with a preparatory phase, including policy dialogue with the state government, identification of nodal government persons for study, development and piloting of study tools, training of research staff, and initiating an umbrella review at specific sites. To identify the burden of stillbirths, a house- to-house survey will identify women who experienced a pregnancy outcome within the past 12 months. Pregnancy outcomes will be documented, specifically live births, abortions, stillbirths, and early neonatal deaths [ENDs] occurring within 24 hours of birth. Additionally, socio-demographic and economic details will be collected, and verbal autopsies will be performed for all women diagnosed with stillbirth. Some sites will also conduct social autopsies, qualitative interviews incorporating a positive deviance method, and minimal tissue invasive sampling [MITS] to explore the reasons for stillbirth in greater depth.

Baseline coverage of antepartum and intrapartum interventions impacting stillbirths will be evaluated at the community, healthcare facility, and health systems levels. Data on coverage indicators will be collected from 900 women either before discharge after delivery or through a community survey of women who experienced a pregnancy outcome in the last three months, using a field-tested tool. The survey will gather information on women’s socio-demographic characteristics, as well as their use of antenatal and intrapartum care.

Healthcare facilities will be assessed for human resources, equipment, medicines, infrastructure [such as labor rooms and laboratories], death reviews conducted, training on maternal and child health programs, and available IEC materials. Deliveries will be observed to assess the quality of intrapartum care, identify any misclassification, and help define stillbirths accurately. The evaluation will examine the availability, quality, and use of stillbirth prevention/safe delivery and care services in healthcare facilities. Standardized tools, including observation checklists, record reviews, and death audit tools, will be used to document these findings. Concurrently, systems-level diagnosis of the potential gaps and bottlenecks in the implementation of various Indian policies, and interventions aimed at reducing stillbirths will be conducted through qualitative interview tools. These methods will engage key stakeholders, pregnant women, and mothers to understand perspectives on existing interventions and assess the availability, quality, and utilization of stillbirth prevention and care services. Furthermore, a time-limited approach, aiming to complete the formative research within three to four months will be considered. Efforts to avoid extending beyond this timeline will be made, unless there is a critical incident severely affecting the data collection process.

The comprehensive analysis will evaluate the processes of existing platforms delivering antenatal and intra-natal care, fidelity of existing policies, assess healthcare providers’ knowledge and skills, explore community knowledge and attitudes, and assess the availability of essential resources. This in-depth systems-level diagnosis will aid in the development of targeted and effective strategies in subsequent study phases.

#### Phase 2: Model development and optimization.

The second phase of the study focuses on two important components of this phase

[a] Co-designing the comprehensive intervention package by the research team and government stakeholders in the workshop model. The initial intervention package is based on insights from the research team’s experience, existing government interventions for safe delivery and the initial literature review is outlined in Table 2. However, this package will be further refined through a comprehensive and systematic examination of the available literature by conducting an umbrella review insight from formative research, and feedback from government authorities.

[b] Optimization of the model for delivering the comprehensive intervention package to deliver the comprehensive intervention package will be optimized in a co-designed strategies with the government stakeholders. The process will be guided by the CFIR framework and will follow a non-linear and recursive implementation process. The model to deliver the intervention package will be optimized through an iterative process and implemented across three levels, i.e., community, health facility, & health systems during pregnancy and childbirth at all seven sites. Adaptive strategies and continuous improvement based on program learning and concurrent evaluation of coverage and quality are deemed critical.

#### Phase 3: Scale-up and monitor the optimized strategy/model.

The research team will implement the optimized model and measure the model’s effectiveness in at least 10 lakh population across the district. In this crucial phase, the government will spearhead the implementation of the refined model across the study areas, closely monitored by the research teams. The coverage and uptake of the intervention package at both community and facility levels will be assessed at endline through a community survey [900 women who have recently delivered], facility assessments, and healthcare worker competency evaluations. This will be compared to the baseline survey. The assessment of intervention coverage and process indicators will be guided by the RE-AIM framework [Reach, Effectiveness, Adoption, Implementation, and Maintenance] as shown in the Table 3.The evaluation of the optimized model’s effectiveness in reducing stillbirth rates will employ a quasi-experimental pre-post design. The research team will work with district and state health teams to strategize the exit of the implementation support. A sustainability plan will also be worked out in consultation with the District Implementation Team. Observation of antenatal check-ups and intra-partum care, delivery observations and monthly record review at ANC clinics, death-review audits and, population-based pregnancy outcome data retrieval from community-level heath staff [ASHAs/ANMs] will be done throughout the three phases. Additionally, the program learning research team will be involved in root-cause analysis of all issues related to inappropriate utilization of care [community-level], and delivery of care [facility level] and will be communicating the findings to implementation support team. This final optimized, evidence based and scalable model will then be handed over to the government for scaling up in the entire district.

**Table 3:** RE-AIM indicators.

Framework domain	Indicators
**Reach**	1.Number of pregnant women [PW] registered during the first trimester. 2. Number of PW receiving four or more “quality” ANC visits: Trimester-wise breakdown of ANC coverage. 3. Number of PW tested for Hemoglobin [Hb] four or more times during respective ANCs. 4. Number of PW tested for blood sugar using OGTT [Oral Glucose Tolerance Test]. 5. Number of PW tested for blood sugar and diagnosed with GDM.
**Effectiveness**	1. Number of PW identified with PIH [Pregnancy-Induced Hypertension]. 2. Number of PW with hypertension managed at the institution. 3. Number of PW with PIH referred to a higher facility. 4. Number of PW identified with for moderate anemia. 5. Number of PW identified with severe anemia. 6. Number of PW with severe anemia managed at the facility. 7. Number of PW with severe anemia referred to a higher facility. 8. Number of PW diagnosed with GDM managed at the facility. 9. Number of PW diagnosed with GDM referred to a higher facility. 10. BOH [Bad Obstetric History] including previous stillbirth- Number of PW with BOH identified, managed at the facility, and referred to higher facilities if necessary.
**Adoption**	1. Number of facilities having in-house ultrasound or linkage to private clinics for USG for pregnant women 2. Number of facilities having required number of dopplers/CTG machines or linkage for required facilities with private facilities 3. Number of facilities having capacity to diagnose GDM and anemia 4. Number of facilities equipped to treat severe anemia 5. Number of facilities where healthcare workers trained on antenatal and intrapartum care
**Implementation**	1. Number of facilities implementing ANC every month in last 12 months under PMSMA 2. Number of facilities using partograph/ safe baby checklist in deliveries 3. Number of facility conducting blood tests for anemia and GDM and gestational hypertension [Hb and OGTT,urine for proteins] 4. Number of facilities managing HRP or complicated intrapartum cases 5. Number of facilities having protocols for management of severe anemia, PIH and GDM

### Date range of participant recruitment

In the formative phase of the study there will not be recruitment of the participant. The recruitment and follow-up of participants will be done in phase-II and III of the study and will range from 1st February 2025 to 31st January 2028.

### Expected outcomes

The main outcome of the current study is the development of a scalable and sustainable model of delivery strategies for implementing the comprehensive intervention package. The secondary outcome is a robust estimation of the burden, timing, and risk factors of stillbirths across all the study sites as well as a reduction in SBR. Additionally, few study sites will try setting up sentinel surveillance for stillbirths.

### Implementation strategies

Implementation strategies are “methods or techniques used to enhance the adoption, implementation, and sustainment of evidence-based practices or programs.” During the pre-initiation phase of this implementation research, we engaged in discussions with the state government and conducted an extensive desk-based literature review to assess the current status of maternal health programs that may impact stillbirth outcomes. In India, multiple barriers contribute to suboptimal birth outcomes, including inadequate utilization of antenatal care [ANC], limited maternal autonomy, inequitable access to care, insufficient awareness of ANC’s importance, and economic constraints that prevent families from prioritizing healthcare. Rural residence further exacerbates these challenges by compounding access issues. The detection and management of high-risk pregnancies [HRP] face additional barriers, such as limited access to sonography, transportation difficulties, and out-of-pocket expenses. Cultural factors, including delayed disclosure of pregnancy until after the first trimester, further impede timely healthcare engagement. Nutritional supplementation programs, delivered through the Integrated Child Development Services [ICDS], encounter challenges such as low awareness of available services, perceptions of poor food quality, and geographic inaccessibility of Anganwadi centers. Intrapartum care is similarly affected by systemic issues, including staffing shortages in labour rooms and gaps in data recording and reporting, which undermine the consistent application of evidence-based practices like the Safe Baby Checklist.

Despite these barriers, facilitators such as increasing levels of parental and maternal education, increased media exposure, and government programs such as the PMSMA and Surakshit Matritva Aashwasan [SUMAN] offer opportunities to enhance maternal healthcare coverage. Interdepartmental collaboration and coordination have also been identified as potential facilitators in improving the identification and care of HRP cases. Additionally, awareness of nutritional services can be enhanced through home visits by Anganwadi workers and targeted health education programs. Training and mentorship initiatives for healthcare providers, coupled with government commitment to quality improvement, are key facilitators for strengthening safe and effective care delivery.

Based on this information, we systematically identified barriers and facilitators using the Consolidated Framework for Implementation Research [CFIR] 2.0. Through the CFIR-ERIC matching tool, we propose Level 1 and Level 2 strategies, grounded in empirical evidence, theoretical models, and pragmatic justifications. We prioritized strategies that, according to a recent systematic review, were associated with positive outcomes in at least 75% of cases [29]. These strategies, particularly in the pre-implementation and implementation phases, have shown strong evidence of supporting successful outcomes. The RE-AIM framework will be used to monitor outcomes across all phases of the study. Our final Implementation Research Logic Model [IRLM] will integrate formative research findings and selected strategies from the ERIC taxonomy (29).Key stakeholders, including healthcare providers and authorities, will be engaged to co-design strategies for adopting and implementing a stillbirth reduction package across seven diverse sites in India. Table 4 presents the preliminary logic model developed using the ERIC-CFIR matching tool, which will be refined based on formative research. Box 1 outlines the principles of the implementation strategies and their potential mode of action.

**Table 4:** Logic model based on ERIC-CFIR.

CFIR Domain	Construct	Barrier/Facilitator	Implementation Level	ERIC Strategy
Outer Setting	**Patient Needs & Resources**	**Barriers**: [i] Economic constraints reduces ANC visits [ii] Transportation challenges. [iii] Out-of-pocket expenses. [iv] Limited access to sonography and specialized care for HRPs due to geographical barriers. [vi] Low literacy and health awareness **Facilitators**: [i] Government programs like PMSMA and SUMAN supporting ANC and HRP services. [ii] Increased media exposure improving awareness about maternal health services. [iii] Community engagement through health workers [ASHAs]	Health System Community Level	[i] Alter incentive/allowance structures under existing schemes [e.g., PMMVY for cash incentive for ANC visits] [ii] Access new funding for HRP services [public private partnership under PMSMA] [iii] Use mass media/Educational activities/Meetings, Materials, Outreach visits to educate mothers about government schemes for transportation, cash transfers etc.
	**External Policies & Incentives**	**Barriers**: [i] Poor coordination between different government programs and healthcare services. **Facilitators**: [i] Government policies [PMSMA, SUMAN] support ANC and HRP detection. [ii] Under the recent POSHYAN Abhiyan increased focus on Supplementary nutrition during pregnancy.	Health System	[i] Engage interdepartmental coordination - to ensure quality antenatal and intraprtum services and benefits of the supplementary nutrition is available to each pregnant women
Inner Setting	**Implementation Climate**	Barriers: [i] Need for staff engagement in quality intrapartum care. [ii] Perceived low quality of ICDS services. [iii] Non-cooperative healthcare staff behaviors and long waiting time in health care facilities [iii] Lack of champions for use of evidence based intra partum protocols Facilitators: [i] Strong government support for ANC and intrapartum care programs.	Facility Level	[ii] Recruit, designate, and train champions [ii]Provide training and ongoing support to improve quality of care and address context specific concerns [like long waiting time, behavior of staff etc.]
	**Available Resources**	Barriers: [i] Manpower shortages in quality intrapartum care. [ii] Geographic access barriers to ICDS services. [iii] Overworked ASHAs. Facilitators: [i] PMSMA and e-PMSMA programmes focus on HRPs and also encourage Obstetricians/ physicians from private sector to provide voluntary services at designated public health facilities	Facility Community Level	[i]Access new funding- [ii] Task shifting/sharing [ii] Adaptability- To overcome geographical barriers to ICDS possibility of community managed SNP for pregnant mothers or other such adaptions may be explored [iii] Use mass media/Educational activities/Meetings, Materials, Outreach visits and educate the community regarding the government schemes especially for HRPs [PMSMA and e PMSMA]
	**Access to Knowledge & Information**	Barriers: [i] Limited knowledge among healthcare workers about HRP risks and Safe Baby Checklist. [ii] ASHAs and healthcare workers unsure about follow-up. [iii] Poor data recording in intrapartum care. Facilitators: [i] Govt programmes like LAQSHYA and DAKSHATA focused on improving quality of services and staff training	Facility	[i]Provide ongoing training and support [ii]Facilitate relay of clinical data to providers and ongoing facilitation
Process	**Engaging**	Barriers [i] Engagement of pregnant women, their families, and frontline healthcare workers in utilizing ANC services, accessing incentives under various schemes, and enhancing the quality of services.	Community Facility Level	[i] Engage community leaders [ii] Conduct educational meetings/SBCC [iii] Asses for readiness
	**Reflecting and Evaluating**	Barriers [i] Poor Adherence to guidelines [for quality ANC care intrapartum care]	Facility and Health System	[i]Audit and provide feedback

Box 1 **Broad principle of Implementation strategies and its description**

**Pre-implementation strategies**

(i) **Conduct educational meetings**: Use mass media and educational materials [Level 1] to raise awareness among women, families, and healthcare providers. Culturally appropriate information in local languages will address misconceptions about ANC services, pregnancy nutrition, and HRP risks. This ensures women understand the importance of timely follow-up, nutritional supplementation, and the Safe Baby Checklist/Evidence based intrapartum care protocols.
(ii) **Conduct local needs assessments**: Identify barriers in different regions to tailor strategies to local needs.
(iii) **Assess for readiness**: Assess the readiness of healthcare facilities, workers, and the health system to implement the Safe Baby Checklist/Evidence based intrapartum care protocols and HRP management protocols, ensuring proper follow-up and referral for each HRP.
(iv) **Identify and prepare champions:** Engage local opinion leaders and community champions [Level 1], such as Anganwadi and community health workers, to promote ANC visits, HRP follow-ups, and nutritional counseling. These champions will educate families about ICDS services and promote the Safe Baby Checklist.

**Implementation strategies:**

(i) **External and internal facilitation**: Provide coaching and support for healthcare workers to effectively use the Safe Baby Checklist, HRP referrals, and ANC services. Training, workshops, and capacity building [Level 1] will equip healthcare staff with skills for managing HRPs delivering appropriate counselling. Support will ensure proper implementation of evidence-based delivery protocols, and workflows may be adjusted to improve intrapartum care.
(ii) **Audit and provide feedback**: Regular **audits and feedback loops** Conduct regular audits and feedback [Level 1] to monitor adherence to the Safe Baby Checklist and evidence-based practices. Feedback will address food quality at Anganwadi centers and monitor HRP referral systems to ensure timely follow-ups. Efficient data management [Level 1] will support communication between healthcare workers and improve care coordination in HRP management.
(iii) **Engage patients and families:** To ensure the involvement of women and their families in decision-making processes related to HRP management and nutritional supplementation.
(iv) **Incentives and funding mechanisms**: Introduce incentives and funding mechanisms [Level 2] to address economic barriers, such as loss of workdays due to ANC visits. Seek additional funding to overcome manpower shortages and transportation challenges, especially for implementing the Safe Baby Checklist and nutritional supplementation. However, all these strategies would be first discussed with the government stakeholders in the co-design workshop and then will be implemented.

### Research team

A multidisciplinary research team will be engaged to support the government partners who will implement the interventions. The implementation research will involve three research teams: the Implementation Support Team [IST], the Program Learning Team [PLT], and the Outcome Monitoring Team [OMT]. The IST will provide technical and managerial support, initially handhold the government partners in effective implementation, assisting with implementation and working closely with all stakeholders. As the government can implement it independently, the support from the IST will gradually be withdrawn. The Program Learning Team [PLT], primarily a qualitative team, will be responsible for the root cause analysis of the barriers/facilitators and the low or high utilization of the interventions. The Outcome Monitoring Team [OMT] will be responsible for independently monitoring predefined outcomes through surveys which are required to assess the impact of the interventions.

### Data analysis

The current study involves qualitative as well as quantitative data collection.The analysis of qualitative data, collected through FGDs and IDIs, will encompass both inductive and deductive approaches. Audio recordings will be transcribed, incorporating field notes into the transcripts. Software packages will facilitate simultaneous data collection and analysis. Responses will be coded using descriptive coding to address research questions, and analytical coding will unveil underlying meanings. Theoretical coding will involve cross-cutting constructs. Grouping codes related to similar themes will form overarching themes and sub-themes. Todetermine the importance of the findings, the key themes will be weighted by estimating the frequency with which they appear and the number of respondents who mention them. A framework approach will be applied, organizing information in a matrix format to examine both cases and themes, ensuring context preservation.

#### Quantitative data.

Real-time quantitative data will be captured using an electronic Data Capture System for monitoring. The research team will extract data from process observations and records, transporting it as CSV files for analysis.Descriptive and inferential statistical analyses will determine associations between risk factors and outcomes, considering statistical significance at p < 0.05 with a 95% confidence interval. Summary statistics, such as means, standard deviations, and proportions, will be calculated for pre and post-intervention phases. Bivariate analysis will assess independent and multiple variable effects on stillbirths. Variables significant in bivariate analysis, clinically relevant, and from previous publications will enter the multivariable analysis model.

### Data quality and management

Training of master trainers from all sites will be conducted at ICMR, to ensure uniformity in understanding the tools and techniques. These master trainers will further train field investigators of respective sites. A training manual, including local terms and simplified definitions, translated into the regional languages will be used. Training of Field investigators on data collection instruments will be performed through off-site and on-site mock surveys. Dedicated software like Redcap etc. will be utilized to collect, store, and monitor the data, which will be handled at the central coordinating unit located at ICMR. The software will be a secure storage system for accurate data collection, organization and statistical analysis. The database will employ a range of consistency checks/validations for clean data collection with minimal errors. Routine reports generated, to monitor data, will be shared with the central team for ongoing adjustment of implementation strategies. Protocol, implementation blueprint and study tools will be developed through a consultative process involving subject experts and district authorities for quality assurance. Fidelity to the study protocol and regular oversight and monitoring of all study activities through regular field visits will also be performed.

### Ethics and dissemination

Ethical approvals have been sought from the Institutional Ethics Committee [IEC] from each of the sites. The written and verbal informed consent process will be obtained from all respondents, adhering to ethical guidelines laid down by the ICMR. The consent form will be translated into the local language taking into consideration the readability score and administered by the interviewer. If the participant is literate, she/he will have the opportunity to read the form of the consent and sign it. However, if the participant is non-literate, the informed consent form will be read aloud by the interviewer in the presence of an impartial literate witness and a thumb impression will be taken. A copy of the signed consent form will be provided to the participants for their records and reference. Data handling and storage will adhere to strict confidentiality and privacy regulations. All personal identifiers will be removed, and data will be anonymized before analysis. Additionally, research team staff and health system staff involved in the study will be trained in good clinical practices [GCP] which is an international ethical and scientific quality standard for the design, conduct, performance, monitoring, auditing, recording, analyses, and reporting of trials and will be standardized for case-records forms, study checklist and data collection and data entry process. Preliminary findings will be communicated to the MOHFW [Ministry of Health and Family Welfare], the respective State Government Health Departments, relevant program officers at the state, district, block levels, community health stakeholders, and ICMR for internal review and feedback. The research team will convene meetings with all stakeholders to deliberate, integrate, and authenticate the findings, culminating in the development of study recommendations. These findings will be showcased during a dissemination event for critical stakeholders. Additionally, they will be shared through policy briefs, national and international scientific gatherings, and peer-reviewed publications.

## Conclusion

The current protocol is a multilevel, multi component intervention study utilizing multiple implementation strategies, which, while reflective of real-life situations, present complexities in measurement [30]. The mixed-method and interdisciplinary approach offers significant insights into strategies for increasing the coverage and uptake of ANC and intrapartum services at both community and facility levels. It has been recommended that in multilevel studies, clearly defining settings, levels of constructs, strategies [including sampling strategies], temporality, and analytical approaches ensures increased rigor in implementation research [31] and we have endeavored to incorporate these elements as applicable in this study. The optimization of implementation strategies, guided by RE-AIM indicators at both system and individual levels, will enhance the transparency, generalizability, and replicability of the intervention package in similar contexts. However, conducting a multilevel study presents significant challenges. These studies are complex, as they require measuring the effects of implementation strategies and interventions on outcomes at various levels, as well as understanding the interaction between these levels [32]. However, potential challenges such as the level of community engagement and its impact on intervention effectiveness, influenced by cultural nuances and community perceptions, as well as the complexity of variables like organizational structures and individual behaviors, are anticipated. Also, due to the shorter period of the study, it may not be possible to evaluate the effectiveness of the model. Despite these challenges, we will design and co-develop an optimized intervention package across seven culturally and geographically diverse sites. This will be valuable for understanding the barriers and facilitators to improving coverage and utilization of antepartum care, as well as strengthening facilities and health systems for maternal care. Additionally, the study will provide insights into which implementation strategies can be used to enhance the uptake of national programs and policies aimed at improving maternal and child health outcomes. This intervention can be of great utility to policymakers and be applied in diverse regions across the country, contributing to broader scalability and impact.
