# Supplementary material for: Implementation research to develop an optimized delivery model for effective implementation of evidence-based interventions to reduce stillbirth in India: A study protocol
Source: PLoS One. 2025 Feb 10;20(2):e0316027. doi: 10.1371/journal.pone.0316027 (PMC11809859; doi:10.1371/journal.pone.0316027)
Supplement: Attachment — Submitted filename: Editorial review_author response_14Nov2024.docx [file pone.0316027.s001.docx]

Dear Jeanelle Pauline P. Cupino
PLOS ONE

We thank you for your response and invitation to submit documents to the PLOS ONE journal. We have taken note of the suggestions and have uploaded all the relevant documents. Kindly find the author response (in blue)

Best Regards

Reema Mukherjee

**Editor’s comments and author responses**

1. Please upload a copy of your trial study protocol as a supporting information file. By the study protocol, we mean the complete and detailed plan for the conduct and analysis of the trial that the ethics committee approved before the trial began. Please send this in the original language. If this is in a language other than English, please also provide a translation. Please detail any deviations from this study protocol in the Methods section of your manuscript. Your study protocol will be made available to the editors and reviewers and will be published as supporting information with your manuscript if accepted for publication. (If you do not agree to this, we will not be able to publish your manuscript). If you have formally published a study protocol for your trial in a journal then you should cite this in your manuscript, but you still need to send us the original document..

**Response**: Thank you for bringing this issue to our attention. The study protocol which has complete plan to conduct the project, methodology, analysis and other relevant details which is approved by the ethics committee before beginning of the trial. The protocol is in English language. As the study is in the preparatory phase and no activities has been initiated, hence no deviations from this study protocol is noted till now. We have uploaded the study proposal as “Study proposal detailed” as 'Other File.'
